# Limited view cone-beam x-ray luminescence tomography based on depth compensation and group sparsity prior

**DOI:** 10.1117/1.JBO.25.1.016004

**Published:** 2020-01-22

**Authors:** Peng Gao, Junyan Rong, Tianshuai Liu, Wenli Zhang, Bin Lan, Xiaoping Ouyang, Hongbing Lu

**Affiliations:** a Fourth Military Medical University, Department of Biomedical Engineering, Xi’an, Shaanxi, China; b Northwest Institute of Nuclear Technology, Xi’an, Shaanxi, China

**Keywords:** x-ray luminescence, limited view, ill-posed, depth compensation, group sparsity

## Abstract

**Significance:** As a promising hybrid imaging technique with x-ray excitable nanophosphors, cone-beam x-ray luminescence computed tomography (CB-XLCT) has been proposed for in-depth biological imaging applications. In situations in which the full rotation of the imaging object (or x-ray source) is inapplicable, the x-ray excitation is limited by geometry, or a lower x-ray excitation dose is mandatory, limited view CB-XLCT reconstruction would be essential. However, this will result in severe ill-posedness and poor image quality.

**Aim:** The aim is to develop a limited view CB-XLCT imaging strategy to reduce the scanning span and a corresponding reconstruction method to achieve robust imaging performance.

**Approach:** In this study, a group sparsity-based reconstruction method is proposed with the consideration that nanophosphors usually cluster in certain regions, such as tumors or major organs such as the liver. In addition, depth compensation (DC) is adopted to avoid the depth inconsistency caused by a limited view strategy.

**Results:** Experiments using numerical simulations and physical phantoms with different edge-to-edge distances were carried out to illustrate the validity of the proposed method. The reconstruction results showed that the proposed method outperforms conventional methods in terms of localization accuracy, target shape, image contrast, and spatial resolution with two perpendicular projections.

**Conclusions:** A limited view CB-XLCT imaging strategy with two perpendicular projections and a reconstruction method based on DC and group sparsity, which is essential for fast CB-XLCT imaging and for some practical imaging applications, such as imaging-guided surgery, is proposed.

## 1 Introduction

X-ray luminescence computed tomography (XLCT) has attracted much attention as a CT/optical dual-mode imaging technique.^1^^–^^3^ It utilizes x-ray excitable nanophosphors as biomarkers to produce visible or near-infrared (NIR) light upon x-ray irritation and naturally combines x-ray structure imaging of high resolution with optical molecular imaging of high sensitivity and specificity. Compared with traditional optical molecular imaging modalities, such as fluorescence molecular tomography (FMT)^4^^,^^5^ and bioluminescence tomography (BLT),^6^^,^^7^ XLCT can detect targets deeply seated in the imaging object with the use of x-rays and provides higher sensitivity due to the avoidance of background optical signals and autofluorescence. With the above advantages, great efforts have been devoted to XLCT imaging and several types of XLCT systems have been proposed according to the x-ray beam shapes. Narrow-beam^2^^,^^3^ and pencil-beam XLCT^8^^,^^9^ can achieve high spatial resolution, but the long imaging time hinders their application to fast biomedical applications. To reduce imaging time, cone-beam XLCT (CB-XLCT) systems^10^^–^^13^ have been proposed for fast imaging to skip the translation step essential in narrow- and pencil-beam XLCT systems.

However, due to high light scattering and low absorption properties in biological tissues, the reconstruction of CB-XLCT is an ill-posed problem. To improve the imaging performance, much prior information has been introduced for CB-XLCT reconstruction, such as permissible region,^14^ CT images,^15^ and sparsity.^16^ Sparsity is the most frequently used prior information for CB-XLCT reconstruction since nanophosphors are usually sparsely distributed inside the body. With this information, in our previous studies, sparse view-based reconstruction methods have been proposed by Gao et al.^16^ and Liu et al.,^17^ where two adjacent targets can be recovered accurately with only four projections evenly distributed in a 360-deg span. Considering this, in some applications, such as intraoperative breast cancer lumpectomy,^18^ only limited view projections could be acquired due to geometry limitation and/or low-dose excitation. It is essential to develop a reconstruction method for the limited view strategy to achieve robust imaging performance in these situations.

Though Liu et al. proposed single-view CB-XLCT reconstruction methods based on sparsity^19^^,^^20^ or group sparsity,^21^ it either works for single target recovery or relies on CT images to obtain group prior, whereas the reconstruction is related to the group LASSO model and imaging performance may be affected by inaccurate group information. In optical imaging, nanophosphors often cluster in some areas, such as tumor regions where luminescent signals are simultaneously nonzero, and rarely distribute in other areas, such as normal tissues where luminescent signals are simultaneously near zero. Thus, beyond sparsity, nanophosphors can also be characterized by group sparsity.^22^ With this prior information, instead of prior CT information, it is possible to improve the image quality of limited view CB-XLCT for multiple targets.

In addition, due to the nonlinear depth sensitivity of measurements detected, the optical intensity of targets far from the detector would be less than those near the detector,^23^^,^^24^ resulting in low reconstructed values for far targets. Therefore, for optical imaging, especially for limited view acquisition, depth compensation (DC) is essential for avoiding depth inconsistency and obtaining high quality reconstructed images.

In this paper, a DC method is first adopted to level off the differences in detection sensitivities by incorporating two weight matrices into the optimization function.^23^ To alleviate the ill-posedness, group sparsity is then introduced as the prior information. Unlike Liu et al. who used the group LASSO model with CT information, here, we add the fused LASSO (FL) penalty to the objective function, which was solved efficiently with a split Bregman iterative method. To evaluate the performance of the proposed method with the DC-FL penalty for CB-XLCT, both numerical simulation and physical phantom experiments were performed and the proposed algorithm was compared with the adaptive Tikhonov (Adaptik),^25^ fast iterative shrinkage-thresholding algorithm (FISTA),^26^ and our previously proposed sparse view-based T-FISTA method^16^ in terms of location error, imaging contrast, shape, and spatial resolution.

The structure of the paper is organized as follows. In Sec. 2, the methodology is presented. First, the forward model for CB-XLCT is briefly described. Second, the DC matrix and FL penalty are formulated. Finally, the split Bregman FL method is described to solve the objective function. In Sec. 3, both numerical simulation and physical phantom experiments are performed to illustrate the performance of the proposed algorithm. In Sec. 4, the results from numerical simulations and physical phantom experiments are presented. In Sec. 5, some discussion and a conclusion are given.

## 2 Methodology

### 2.1 Forward Problem

In the CB-XLCT system, x-rays penetrate the imaging object based on Lambert–Beers’ law, and the x-ray intensity ($W\;{\mathrm{cm}}^{-3}$) distribution X(r) in the imaging object is expressed as follows:^10^

$$X(r)=X(r_{0})\exp\{-\int_{r_{0}}^{r}\mu_{t}(\tau )d\tau \},$$ (1)

where $X(r_{0})$ is the intensity of x-ray ($W\;{\mathrm{cm}}^{-3}$) at the initial position $r_{0}$, and $\mu_{t}(\tau )$ is the x-ray attenuation coefficient (${\mathrm{cm}}^{-1}$) obtained from x-ray transmission data. When irradiated by x-rays, nanophosphors distributed in the imaging object can emit visible or near-infrared (NIR) light, which is expressed as follows:

S(r)=εX(r)n(r), (2)

where S(r) is the source energy density ($W\;{\mathrm{cm}}^{-3}$), ε is the light yield defined as the quantum yield per unit nanophosphor concentration ($\mathrm{mg}\;{\mathrm{mL}}^{-1}$), and n(r) is the nanophosphor concentration in position r. The light transportation in scattering media is modeled by the radiative transfer equation (RTE), but solving the RTE directly is extremely difficult. Considering the highly scattering and weakly absorbing properties of biological tissues in the visible or NIR spectral window, the RTE model is simplified to the following diffusion equation model:^7^

$$\left\{ \begin{array}{ll} -\nabla [D(r)\nabla \Phi (r)]+\mu_{a}(r)\Phi (r)=X(r) & \left(r\in \Omega \right)\\ \Phi (r)+2\alpha D(r)[\nu \nabla \Phi (r)]=0 & \left(r\in \partial \Omega \right) \end{array},\right.$$ (3)

where D(r) is the diffusion coefficient that is calculated by $D(r)={\left[3[\mu_{a}(r)+\mu_{s}^{\prime }(r)]\right]}^{-1}$ and $\mu_{a}$ and $\mu_{s}^{\prime }$ are the absorption and reduced scattering coefficients of the tissue, respectively. Φ(r) is the photon fluence at position r, Ω is the domain of the imaged object, ∂Ω denotes the boundary of Ω, α is a factor describing the optical reflective index mismatch, and ν is the outward unit normal vector on ∂Ω.

With the finite-element method, Eq. (3) is discretized into a matrix equation, which builds a linear relationship between the nanophosphor concentration N and photon measurements on the object surface $\Phi_{\mathrm{meas}}$:

$$\mathrm{AN}=\Phi_{\mathrm{meas}},$$ (4)

where A is a weight matrix used to map the unknown nanophosphor distribution to known measurements.

### 2.2 Limited View Reconstruction with DC-FL for CB-XLCT

**Fig. 1:**
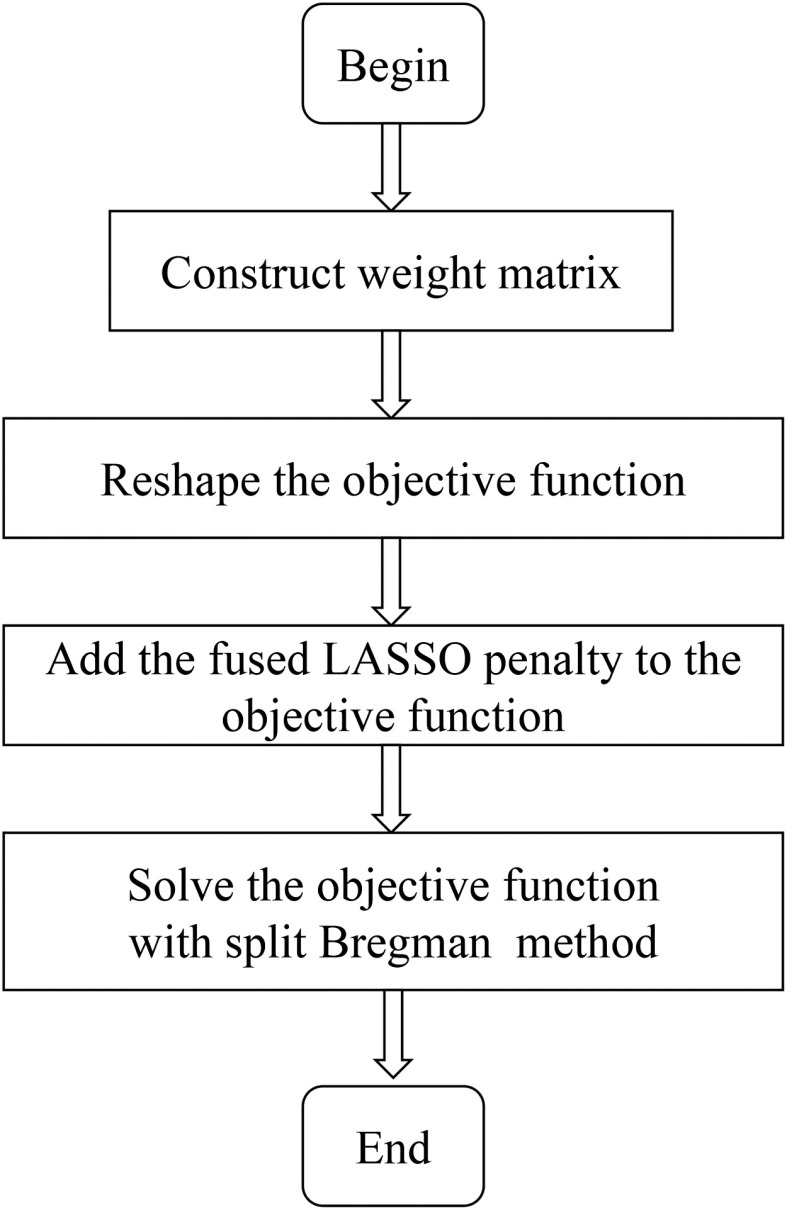
The flowchart of the proposed DC-FL algorithm.

It is known that the detection sensitivity in optical imaging decreases nonlinearly with increased depth.^24^ This makes CB-XLCT measurements hypersensitive to targets near the detector, which typically happens in limited view imaging of multiple targets. The ill-posed weight matrix will lead to reconstructed biases toward the superficial targets. To level off this bias, DC is first implemented by incorporating two weight matrices into the objective function. Then, to take advantage of the priors in CB-XLCT, a FL penalty is added for reconstruction based on group sparsity priors. The final objective function is solved with the split Bregman method considering its computation efficiency. Figure 1 gives the flowchart of the proposed algorithm.

#### 2.2.1 Depth compensation for the inverse problem

Generally, the weight matrix A is ill-posed, and it is impractical to solve N directly. To obtain a unique and stable solution, Eq. (4) is solved by the following minimization problem with the $L_{p}$ regularization term:

$$\underset{n}{\min}\{{\left\|\mathrm{AN}-\Phi_{\mathrm{meas}}\right\|}^{2}+\lambda {\left\|N\right\|}_{P}\},$$ (5)

where λ is the regularization parameter and ${\left\|N\right\|}_{p}$ is the $L_{p}$-norm of N.

For effective DC, two weight matrices, i.e., data weight matrix $W_{d}$ and model weight matrix $W_{m}$ are introduced,^23^ and Eq. (5) are further converted to the following equations:

$$\left\{ \begin{array}{l} \underset{y}{\min}\{{\left\|{\mathrm{AW}}_{d}W_{m}^{-1}Y-\Phi_{\mathrm{meas}}\right\|}_{2}^{2}+\lambda {\left\|Y\right\|}_{P}^{2}\}\\ Y=W_{m}N \end{array}.\right.$$ (6)

In detail, $W_{d}$ provides constraints or *a priori* information for the solution, which is constructed as

$$W_{d}=\mathrm{diag}(\overset{˜}{N})=(\begin{array}{ccc} \overset{˜}{n_{1}^{q}} & \cdots & 0\\ \vdots & \ddots & \vdots \\ 0 & \cdots & \overset{˜}{n_{n}^{q}} \end{array}),$$ (7)

where $\overset{˜}{N}$ is a rough approximation to the true solution N and q controls the compromise between a close fit to the data and the stability of the solution, which was set according to Ref. 24. In this paper, the rough approximation is obtained by the T-FISTA method.

$W_{m}$ is used to level off the differences in detection sensitivities and is given by

$$\left\{ \begin{array}{l} \beta_{j}=\frac{1}{\left|\max(A_{j})-\min(A_{j})\right|}\\ W_{m}=\mathrm{diag}\{\beta_{j}*{\left\|A_{j}\right\|}_{2}^{-1}\}=(\begin{array}{ccc} \beta_{j}*{\left\|A_{j}\right\|}_{2}^{-1} & \cdots & 0\\ \vdots & \ddots & \vdots \\ 0 & \cdots & \beta_{n}*{\left\|A_{n}\right\|}_{2}^{-1} \end{array}),\;j=1,2,\cdots ,N \end{array},\right.$$ (8)

where $\beta_{j}$ is a normalization factor that is inversely proportional to the absolute largest difference between the elements within each column.

#### 2.2.2 Fused LASSO penalty for the inverse problem

In this paper, the group sparsity of the luminescent signals, which means that the luminescent signals should be roughly piecewise constant, is introduced as *a priori* information. As a result, the FL penalty is incorporated into Eq. (6) to make use of the group sparsity:

$$\underset{y}{\min}\{{\left\|{\mathrm{AW}}_{d}W_{m}^{-1}Y-\Phi_{\mathrm{meas}}\right\|}_{2}^{2}+\lambda_{1}{\left\|Y\right\|}_{1}^{2}+\lambda_{2}{\left\|RY\right\|}_{1}^{2}\},$$ (9)

where

$$R_{ij}=\{\begin{array}{ll} -1 & j=i,i=1,2,\cdots \\ 1 & j=i+1,i=1,2,\cdots \\ 0 & \text{otherwise} \end{array}.$$ (10)

The regularization term with parameter $\lambda_{1}$ encourages the sparsity of the reconstructed signals, while the other term with parameter $\lambda_{2}$ shrinks the differences between neighboring signals.

#### 2.2.3 Split Bregman method for the fused LASSO problem

Generally, solving Eq. (9) is computationally challenging due to the nondifferentiability of the objective function. Here, in this paper, we adopted a method based on the split Bregman iteration for solving the general FL problem.^27^ The Bregman iteration technique is based on the Bregman distance that generalizes the concept of metric associating a distance to a convex function not necessarily differentiable, and it addresses the objective problem by analyzing it into several functions and minimizing them separately in an efficient simple way. We introduce two auxiliary variables, a and b, to represent Y and RY, and u and v are dual variables corresponding to the linear constraints Y=a and RY=b, respectively. The split Bregman iteration can be implemented as follows:

Initialization: $y^{0}$, $a^{0}$, $b^{0}$, $u^{0}$, and $v^{0}$.
Let $V(y)={\left\|{\mathrm{AW}}_{d}W_{m}^{-1}Y-\Phi_{\mathrm{meas}}\right\|}_{2}^{2}$
**Repeat:**
1) $y^{m+1}=\arg\;\underset{y}{\min}\{V(y)+\langle u^{m},y-a^{m}\rangle +\langle v^{m},Ry-b^{m}\rangle +\frac{\mu_{1}}{2}{\left\|y-a^{m}\right\|}_{2}^{2}+\frac{\mu_{2}}{2}{\left\|Ry-b^{m}\right\|}_{2}^{2}\}$
2) $a^{m+1}=\zeta_{u_{1}^{-1}\lambda_{1}}(y^{m+1}+\mu_{1}^{-1}u^{m})$
3) $b^{m+1}=\zeta_{u2^{-1}\lambda_{2}}(Ry^{m+1}+\mu_{2}^{-1}v^{m})$
4) $u^{m+1}=u^{m}+\delta_{1}(y^{m+1}-a^{m+1})$
5) $v^{m+1}=v^{m}+\delta_{2}(Ry^{m+1}-b^{m+1})$ where ζ a soft thresholding operator.
**Until**
Convergence

In this paper, the regularization parameters $\lambda_{1}$ and $\lambda_{2}$ were set to 0.1 and 1, respectively. The iteration numbers were set to 150 in simulations and 300 in phantom experiments according to the results, respectively. More detailed information on this method can be found in Ref. 28.

## 3 Experimental Setup

### 3.1 Numerical Simulation Setup

**Fig. 2:**
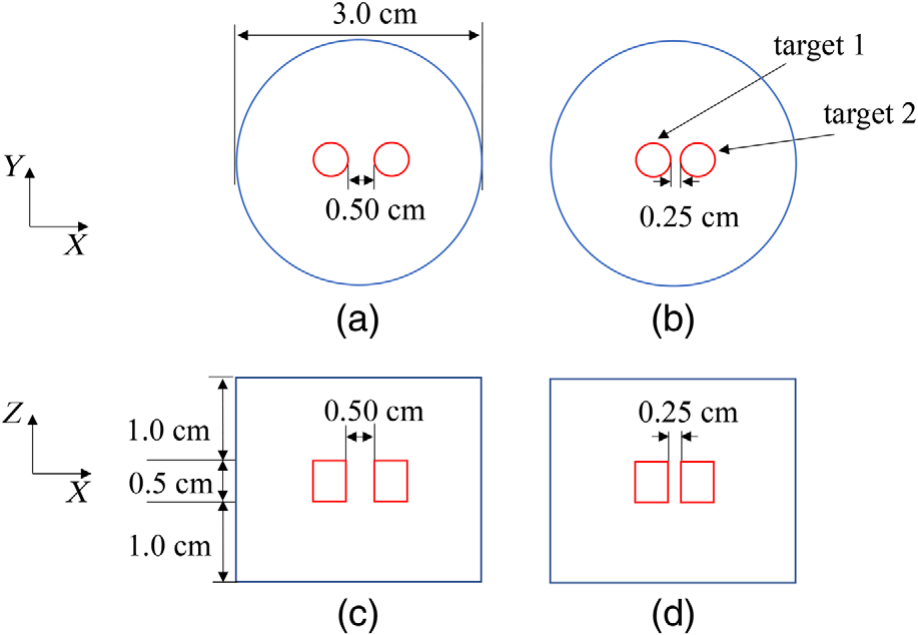
Setup for the cylinder phantom. (a), (b) The view of the XY plane of the two cases. (c), (d) The view of the XZ plane of the two cases. Two luminescent targets of $Y_{2}O_{3}:{\mathrm{Eu}}^{3+}$ were placed inside a cylinder phantom. The EED along the X axis is 0.30 cm in case 1 (first column) and 0.10 cm in case 2 (second column).

**Fig. 3:**
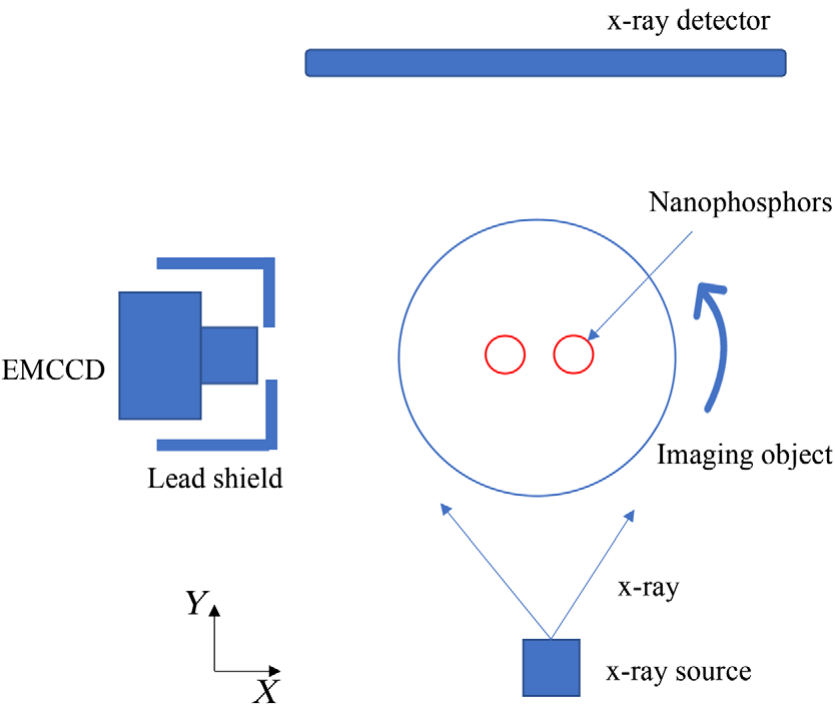
Schematic diagram of the hybrid CB-XLCT/XCT imaging system and the initial phantom position setup used in simulation and phantom experiments.

Numerical simulations were implemented with a cylinder phantom with two luminescent targets embedded to evaluate the performance of the proposed method. The geometry configuration of the phantom is shown in Fig. 2. It was a cylinder placed on a rotating stage, with the rotational axis defined as the Z axis and the bottom plane set as Z=0  cm. The diameter and height of the cylindrical phantom were 3.0 and 2.5 cm, respectively. To approximate high scattering media, the phantom was filled with 1% intralipid, where the absorption coefficient and reduced scattering coefficient were set to 0.02 and $10\;{\mathrm{cm}}^{-1}$, respectively. Two luminescent targets with nanophosphors of $Y_{2}O_{3}:{\mathrm{Eu}}^{3+}$ were placed in the phantom at a depth of 1.0 cm. The targets were 0.4 cm in diameter and 0.5 cm in height. The edge-to-edge distance (EED) of the two targets was set to 0.30 cm in case 1 and 0.10 cm in case 2. The nanophosphors were excited by CB x-rays with a tube voltage of 40 kV. The initial phantom position setup is shown in Fig. 3. Luminescent images were collected at every 90 deg for a full span of 360 deg. A total of four projections were collected, some of which were used for reconstruction. The detectors located on finite-element nodes of the boundary were inside 160 deg. To make the simulations more realistic, white Gaussian noise was added to generate noisy boundary measurements with a signal-to-noise ratio set to 20 dB. In this paper, the analytical simulation was conducted with COMSOL Multiphysics 3.3 (COMSOL, Inc., Burlington, Massachusetts).

### 3.2 Phantom Experiments Setup

**Fig. 4:**
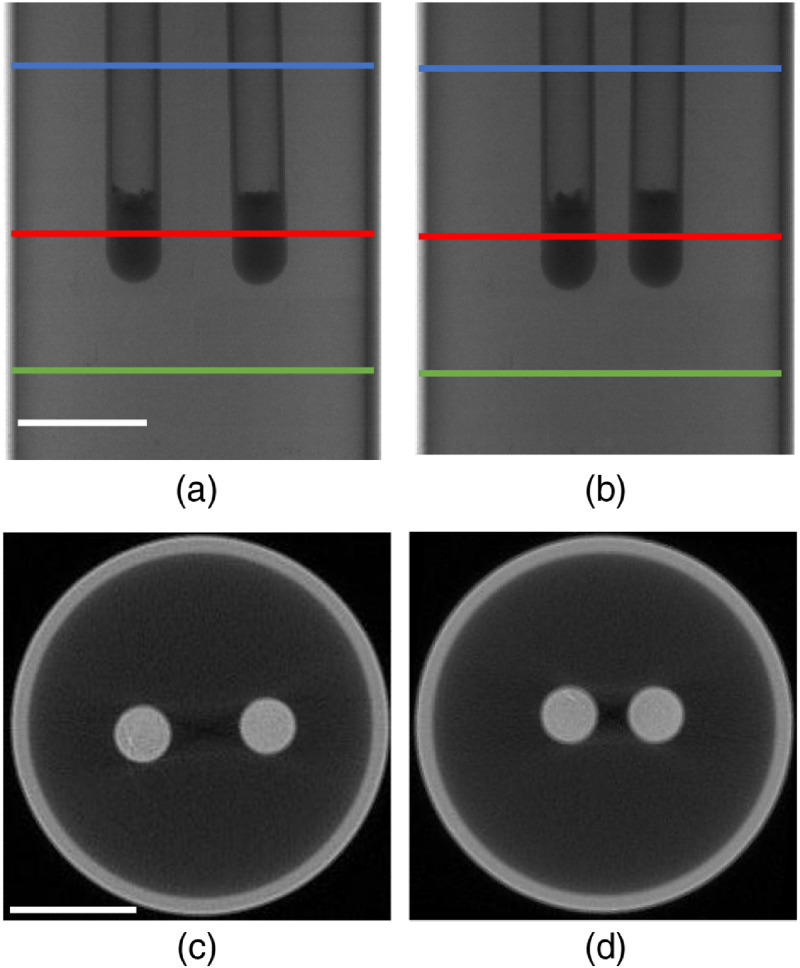
Setup for experimental studies. (a), (b) Representative x-ray projections of the phantoms with EED=0.50  cm and EED=0.23  cm, respectively. Regions between the blue and green lines are used for analysis. (c), (d) XCT slice of the phantom, corresponding to the slice indicated by the red line in (a) and (b), respectively. The left and right tubes are named as tube 1 and tube 2, respectively. White scale bars in (a) and (c): 1 cm.

A physical phantom similar to the simulations was imaged based on the custom-made CB-XLCT system developed by our lab. The system consists of a microfocus CB x-ray source that is used to excite the phantom, a highly sensitive electron-multiplying charge-coupled device (EMCCD) adopted for luminescent projections collection, and an x-ray flat panel detector to collect transmitted x-ray signals, as described in Fig. 3. Figure 4 illustrates the setup of the physical phantom. It was a transparent glass cylinder with a diameter of 3.0 cm and a height of 7.0 cm, filled with 1% intralipid and water (with an absorption coefficient of $0.02\;{\mathrm{cm}}^{-1}$ and a reduced scattering coefficient of $10\;{\mathrm{cm}}^{-1}$). Two glass tubes of 0.4 cm diameter that contained nanophosphors of $Y_{2}O_{3}:{\mathrm{Eu}}^{3+}$ were implanted in the phantom. The concentration of $Y_{2}O_{3}:{\mathrm{Eu}}^{3+}$ was $0.1\;g\;{\mathrm{mL}}^{-1}$. Similar to simulation studies, two cases of phantom experiments with different EEDs (0.50 cm in case 1 and 0.23 cm in case 2) were performed. The detectors located on finite-element nodes of the boundary were inside 160 deg. The imaging details can be found in Ref. 16.

Figures 4(a) and 4(b) show the representative x-ray projections of the physical phantoms with different EEDs. Representative CT slices of the phantoms with different EEDs are shown in Figs. 4(c) and 4(d). Regions between the blue and green lines were selected for the study.

### 3.3 Quantitative Evaluation

To quantitatively evaluate the performance of the proposed reconstruction method, in this paper, three state-of-the-art methods were implemented for comparison, including the adaptive Tikhonov regularization (Adaptik, L-2 norm), FISTA (L-1 norm), and our previously proposed revised T-FISTA method. The regularization parameters were set according to Ref. 24, respectively. The iteration numbers were chosen empirically according to the results and to ensure the convergence of the calculation.

Images reconstructed by different methods were compared in terms of location error, imaging contrast, shape, and spatial resolution. For quantitative evaluation, the position error (PE), Dice similarity coefficient (DICE), contrast-to-noise ratio (CNR), and spatial resolution index (SPI) were calculated, respectively.^16^

PE is defined as the Euclidean distance between the actual and reconstructed luminescent positions:

$$\mathrm{PE}={\left\|p_{r}-p_{t}\right\|}_{2},$$ (11)

where $p_{r}$ and $p_{t}$ denote the centers of the reconstructed and true targets, respectively.

DICE evaluates the similarity between the actual and reconstructed luminescent areas:

$$\mathrm{DICE}=\frac{2|{\mathrm{ROI}}_{r}\cap {\mathrm{ROI}}_{t}|}{\left|{\mathrm{ROI}}_{r}|+|{\mathrm{ROI}}_{t}\right|},$$ (12)

where ${\mathrm{ROI}}_{r}$ and ${\mathrm{ROI}}_{t}$ denote the reconstructed and true luminescent areas, respectively. The closer the reconstruction is to the true target, the closer the DICE is to 1. Otherwise, the DICE is closer to 0.

CNR is used for quantitative evaluation of image contrast:

$$\mathrm{CNR}=\frac{\left|{\text{Mean}}_{\mathrm{ROI}}-{\text{Mean}}_{\mathrm{BK}}\right|}{{\left(w{\mathrm{Var}}_{\mathrm{ROI}}^{2}+(1-w){\mathrm{Var}}_{\mathrm{BK}}^{2}\right)}^{1/2}},$$ (13)

where the subscripts ROI and BK denote the target and background regions, respectively, mean and var denote the mean value and variance, respectively, and w is the weighting factor calculated by the relative volumes of the ROI.

SPI is a spatial resolution quantitative index to analyze the performance of the algorithms in resolving two targets:

$$\mathrm{SPI}=\frac{n_{\max}^{l}-n_{\text{valley}}^{l}}{n_{\max}^{l}-n_{\min}^{l}},$$ (14)

where $n_{l}$ denotes the value of the profile along a given line that connects the two centers on the reconstructed cross-section. $n_{\max}^{l}$, $n_{\min}^{l}$, and $n_{\text{valley}}^{l}$ are the maximal, minimal, and valley value between the two peak values, respectively. The more clearly the two targets are separated, the closer the SPI is to 1.

## 4 Results

### 4.1 Numerical Simulations

**Fig. 5:**
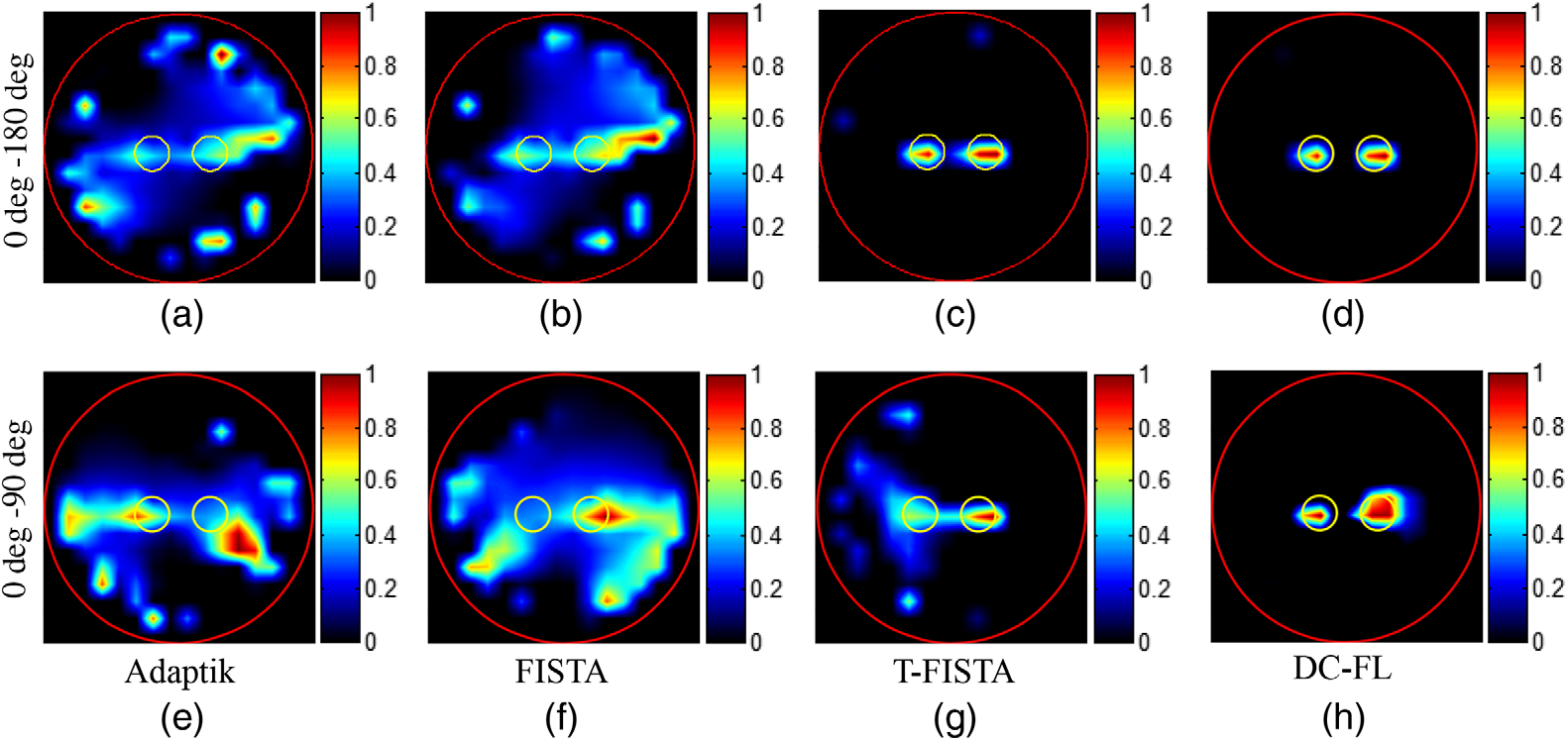
CB-XLCT reconstruction results of simulation case 1 (EED=0.30  cm). (a)–(h) The first to the fourth column are results obtained with the Adaptik, FISTA, T-FISTA, and DC-FL. The first and second row are the results reconstructed with two projections of 180-deg and 90-deg view spans, respectively. The yellow circles depict the true positions of the two targets, and the red circles depict the boundary of the phantom. All images are normalized to the maximal value.

Figure 5 shows the reconstruction results of case 1 with two projections in numerical simulations. The height of the cross-section is 1.3 cm. The red circle in each image depicts the phantom boundary, and the two yellow circles depict the true positions of the two targets. Two groups of reconstructions were conducted. In the first group, the projection pair of 0 deg to 180 deg was adopted for reconstruction, and the results are shown in the first row. It can be seen that both the traditional L2-norm based method Adaptik and the L1-norm based method FISTA could not resolve the two targets and introduce more noise in the images. Though the T-FISTA method could resolve the two targets clearly, some noise existed in the image when compared with that obtained by the proposed method. In the second group, a reduced imaging span was adopted and the projection pair of 0 deg to 90 deg was used for reconstruction. The results are shown in the second row of Fig. 5. In this group, the other three methods could not resolve two targets effectively, while the proposed algorithm could do it with good image quality.

**Fig. 6:**
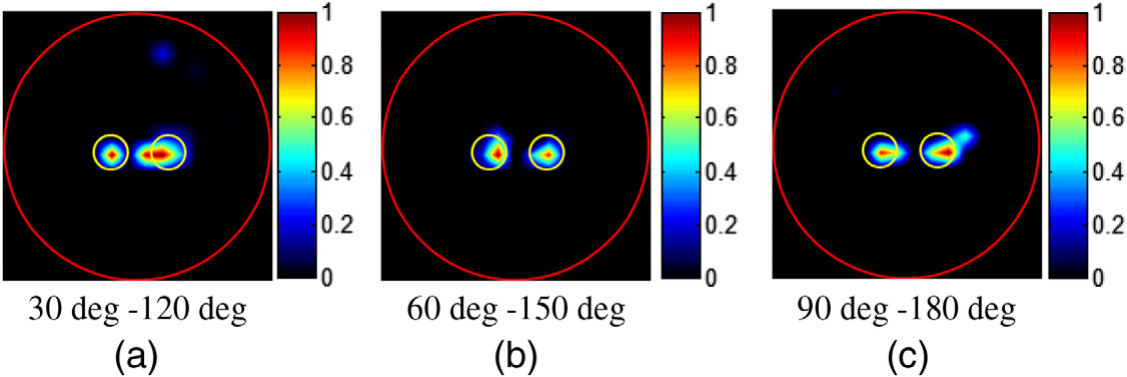
The reconstruction results for illustrating the effect of the initial position of the 90-deg span. (a)–(c) Three different 90-deg span projection pairs: (a) 30 deg to 120 deg, (b) 60 deg to 150 deg, (c) and 90 deg to 180 deg.

To demonstrate whether the initial position of the 90-deg span would affect the imaging performance, we performed another numerical simulation, where three other 90-deg span projection pairs, 30 deg to 120 deg, 60 deg to 150 deg, and 90 deg to 180 deg, were used, respectively. The results are shown in Fig. 6. It can be seen that, no matter where the projection started, the quality of reconstructed images was quite similar. This indicates that the initial position of the limited view span has little effect on the reconstruction results.

**Fig. 7:**
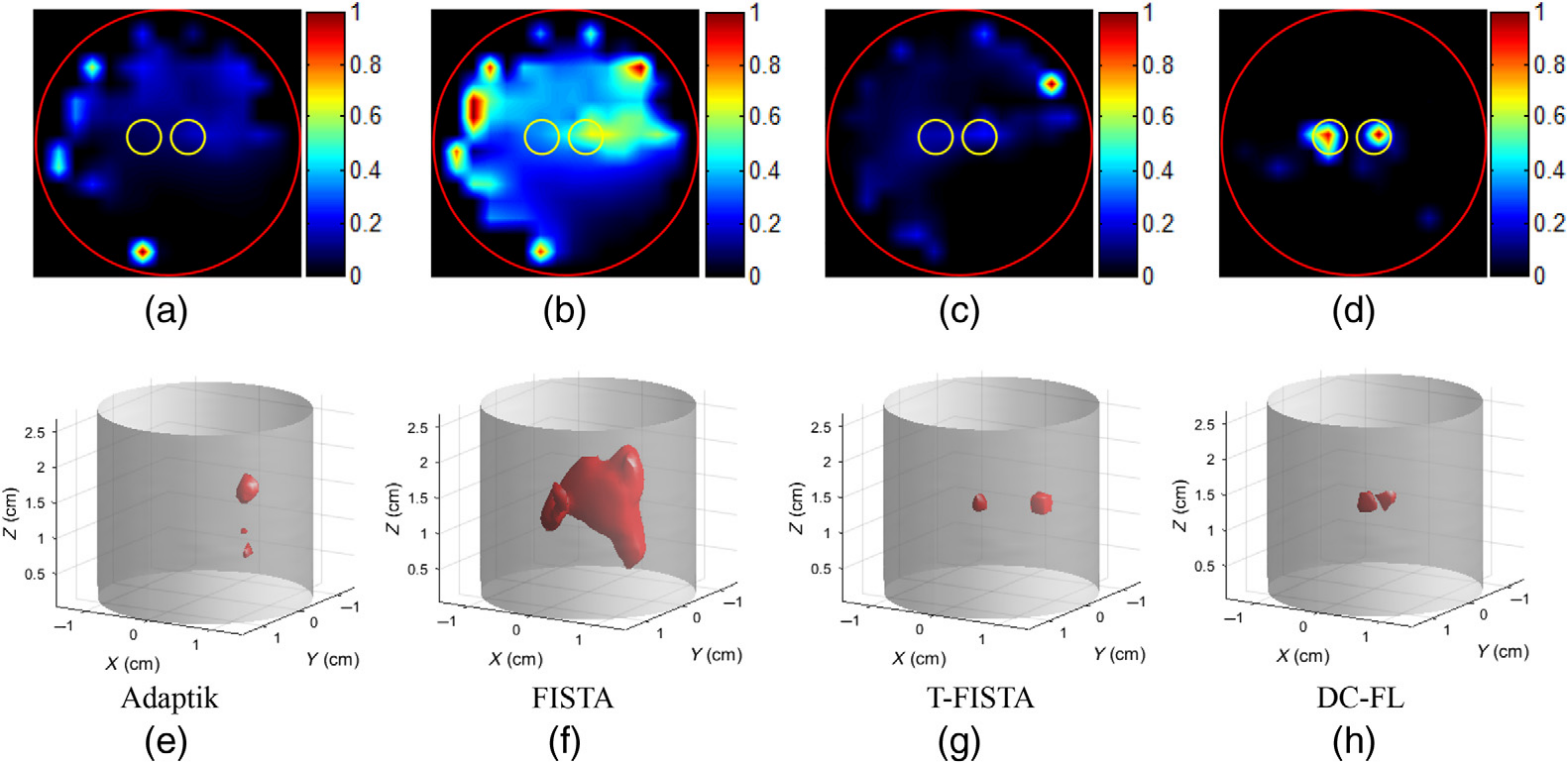
(a)–(h) CB-XLCT reconstruction results of simulation case 2 (EED=0.10  cm). From left to right: results obtained by the Adaptik, FISTA, T-FISTA, and DC-FL, respectively. From top to bottom: 2D and 3D results reconstructed with two projection pairs of 0-deg to 90-deg view span, respectively. The cylinders depict the phantom, and the red objects represent the luminescent targets.

Figure 7 shows the reconstruction results of simulation case 2, where two projection pairs of 0 deg to 90 deg were used. The upper row in Fig. 7 gives 2D slices while the lower row shows corresponding 3D visualization results. In 3D results, values of <10% of the maximum value were neglected. The cylinders depict the phantom, and the red objects represent the luminescent targets. We can see that, as the two targets get closer, the tomographic images obtained with the first three methods [Figs. 5(a)–5(c)] become much worse than those in case 1, and none of these methods could separate the two targets clearly. By contrast, the proposed DC-FL method could achieve high-quality imaging with accurate position and less noise, which can be further demonstrated by the 3D renderings.

To further evaluate the performance of the proposed method, quantitative analysis was performed, as shown in Table 1, which corresponds to the upper row in Fig. 7. The proposed method achieves the least PE, which means the reconstructed positions of the two targets were most accurate compared with those obtained by the other three methods. In addition, the proposed method yields the highest DICE, CNR, and SPI, indicating that the targets were recovered with the best shape, least noise, and best spatial resolution.

**Table 1:** Quantitative analysis of simulation case 2 with two projection pairs of 0 deg to 90 deg.

	PE (mm)		DICE		CNR	SPI
	Tube 1	Tube 2	Tube 1	Tube 2		
Adaptik	3.79	2.97	0.07	0.06	0.70	0.37
FISTA	3.81	3.05	0.05	0.05	1.40	0.30
T-FISTA	5.94	0.82	0.06	0.06	1.84	0.23
DC-FL	0.22	0.29	0.82	0.73	8.38	0.94

### 4.2 Phantom Experiments

**Fig. 8:**
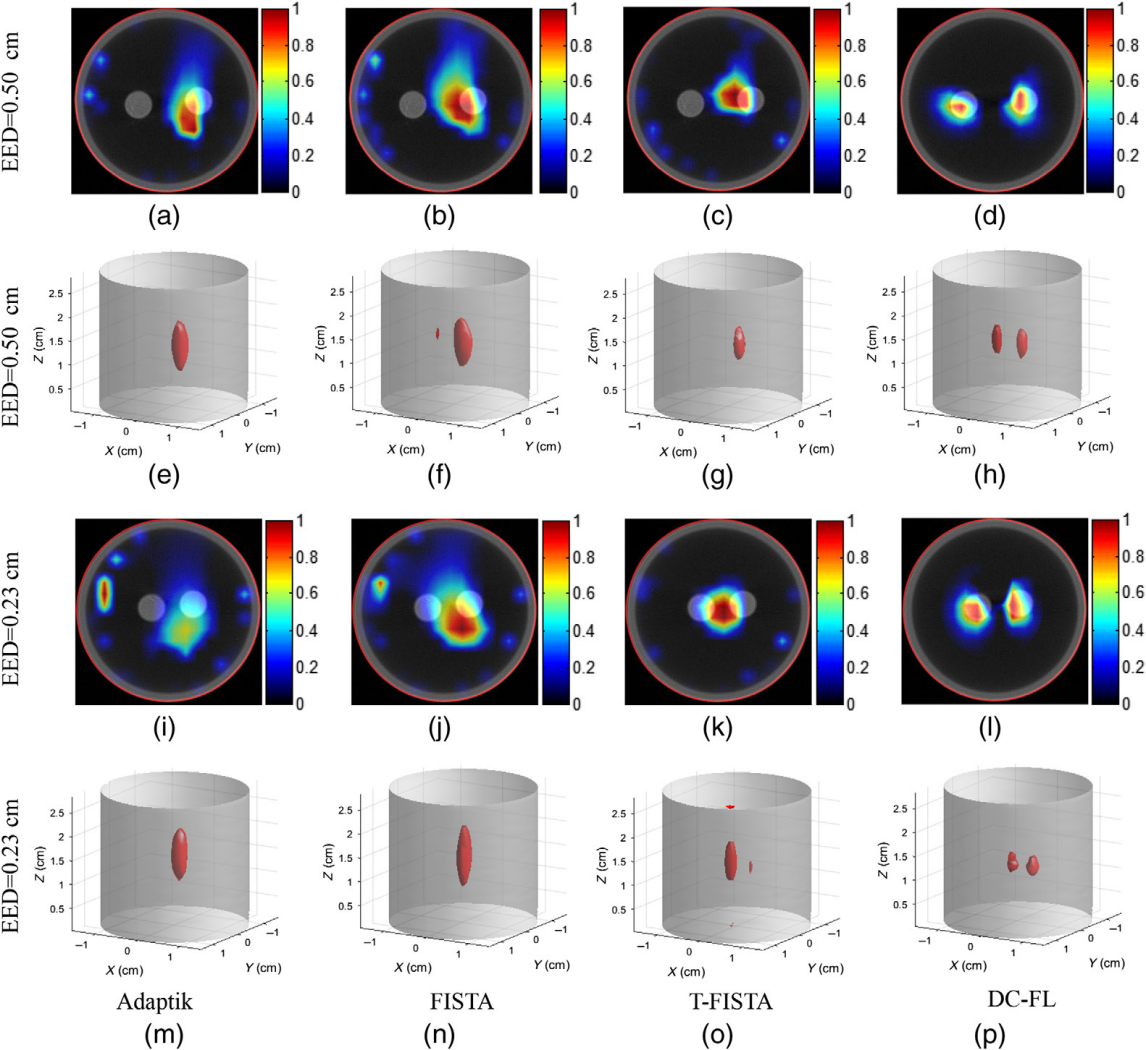
Results of the phantom experiment. The first and third row shows the tomographic fused XLCT/CT images, while the second and fourth row depicts the XLCT 3D visualization results of (a)–(h) case 1 (EED=0.50  cm) and (i)–(p) case 2 (EED=0.23  cm), respectively. Reconstructions obtained by the Adaptik, FISTA, T-FISTA, and DC-FL are shown from the first to the fourth column.

Figure 8 shows the cross-sectional and 3D results of the phantom experiment. The first and third rows show the tomographic fused XLCT/CT images corresponding to the CT slice indicated by the red line in Fig. 4(a), while the second and fourth rows depict the XLCT 3D visualization results of case 1 (EED=0.50  cm) and case 2 (EED=0.23  cm), respectively. Reconstructions obtained by the Adaptik, FISTA, T-FISTA, and DC-FL are shown from the first to the fourth column. In both cases, two projection pairs of 0 deg to 90 deg were adopted for reconstruction.

The phantom experimental results indicate that the limited view used in the reconstruction could only resolve one target [Figs. 8(a)–8(c) and 8(i)–8(k)] with the Adaptik, FISTA, and T-FISTA methods, which might be due to one target being closer to the detector than the other in the two projections. With the DC-based FL reconstruction strategy, both targets could be resolved accurately as expected [Fig. 8(a)], even when the two tubes were close with an EED of 0.23 cm [Fig. 8(l)]. The performance was further confirmed by the 3D results [Figs. 8(h) and 8(p)].

**Fig. 9:**
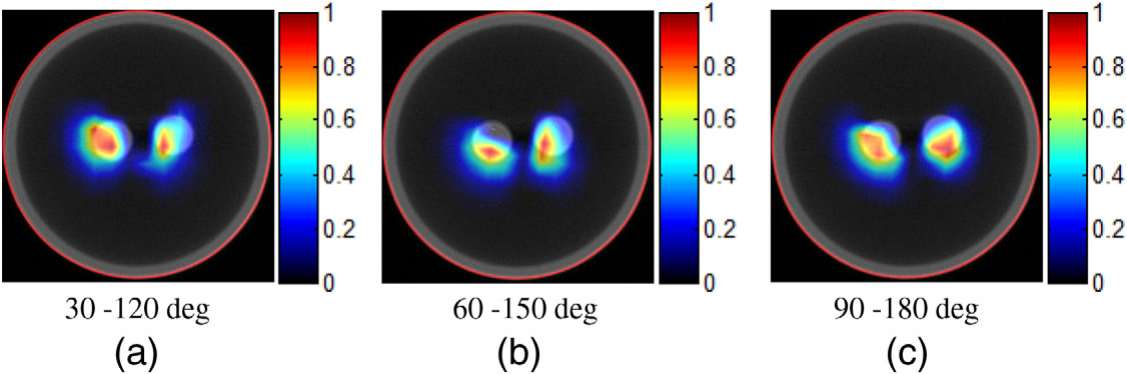
Results of the phantom experiment case 2 with different 90-deg span projection pairs. (a)–(c) Three different 90-deg span projection pairs: (a) 30 deg to 120 deg, (b) 60 deg to 150 deg, and (c) 90 deg to 180 deg.

To demonstrate whether the initial position would affect the imaging performance of the phantom experiments, three other 90-deg span projection pairs, including 30 deg to 120 deg, 60 deg to 150 deg and 90 deg to 180 deg were tested, respectively. As shown in Fig. 9, images reconstructed from different projection pairs are quite similar, indicating that the initial position of the limited view span has little effect on the reconstruction results.

To further validate the proposed algorithm, a quantitative evaluation of phantom experiment 2 (third row in Fig. 8) was carried out, as presented in Table 2. Similar to results in simulations, this demonstrates that, compared with the other methods, the proposed method yields the smallest LE and the highest CNR, DICE, and SPI, indicating that the targets were recovered with the least relative error.

**Table 2:** Quantitative analysis of phantom results of case 2.

	PE (mm)		DICE		CNR	SPI
	Tube 1	Tube 2	Tube 1	Tube 2		
Adaptik	5.65	1.33	0.01	0.23	1.68	0.24
FISTA	4.85	3.44	0.11	0.17	1.91	0.10
T-FISTA	3.52	3.45	0.28	0.26	2.78	0.02
DC-FL	0.93	0.64	0.48	0.68	5.00	0.98

## 5 Discussion and Conclusion

In this study, we established a group sparsity-based limited view CB-XLCT reconstruction method. To reduce the depth-dependent value inconsistency caused by the limited view strategy, DC was first adopted. Then, the group sparsity was introduced as prior information in consideration of nanophosphors usually being clustered in groups. Both numerical simulations and phantom experiments validate the performance of the proposed method.

The reconstruction results of numerical simulations and phantom experiments show that, with the proposed method, we can resolve the distribution of the nanophosphors even when the targets are close to each other, using only two projections at 0 deg and 90 deg (Figs. 6–8). Quantitative analysis (Tables 1 and 2) together with the 3D renderings indicates that, compared with Adaptik, FISTA, and T-FISTA methods, the proposed DC-FL method achieved the highest location accuracy, contrast, and resolution and the most consistent shape. All of these results demonstrate the potential of the proposed method in improving imaging performance and reducing the imaging time and radiation dose of limited view CB-XLCT. Though DC-FL is designed for CB-XLCT in this paper, it can be extended to other XLCT systems, such as narrow-beam XLCT or fan-beam XLCT systems. Further, with appropriate forward models, DC-FL can be applied to other optical reconstructions, such as FMT and BLT.

Our results indicate that no matter which projection is used as the initial view (0 deg, 30 deg, 60 deg, or 90 deg), the imaging performance is similar (Fig. 6), which suggests that 90-deg span could be a robust imaging strategy for limited view imaging. We also find that a smaller span (single view in simulations or 60 deg in phantom experiments) may also achieve comparable performance. However, this depends on the position of the initial view, which may lead to unstable results. In addition, the imaging cost has been greatly reduced, i.e., from 94 s (with four projections evenly collected in a 360-deg span) to 17 s (with two perpendicular projections collected in a 90-deg span) in our experiments.

Although the proposed method achieved better performance, some important issues need to be further addressed. In this work, all of the hyperparameters including regularization parameters, iteration numbers, and initial values were empirically optimized based on value ranges suggested by references. The automatic selection of these parameters would substantially benefit CB-XLCT. In addition, the rough approximation of the solution used for constructing $W_{d}$ is critical for the imaging performance of the proposed method. The more accurate the approximation is, the better the result that can be achieved. Furthermore, effective optimization methods used to solve the FL problem, such as the primal-dual Newton conjugate gradient method,^29^ may further improve the limited view imaging. In future work, more effort will be devoted to resolving more targets with smaller EED by the proposed method.
